# Supplementary material for: Tree inference for single-cell data
Source: Genome Biol. 2016 May 5;17:86. doi: 10.1186/s13059-016-0936-x (PMC4858868; doi:10.1186/s13059-016-0936-x)
Supplement: Additional file 1 — Supplementary figures, a supplementary table, and a description of the additional MCMC moves and their effect on convergence. (PDF 970 kb) [file 13059_2016_936_MOESM1_ESM.pdf]

Supplementary material for
"Tree inference for single-cell data"

Katharina Jahn[1,2,⋆], Jack Kuipers[1,2,∗], and Niko Beerenwinkel[1,2]

[1] Department of Biosystems Science and Engineering, ETH Zurich, Basel, Switzerland
[2] SIB Swiss Institute of Bioinformatics, Basel, Switzerland

Supplementary Figures

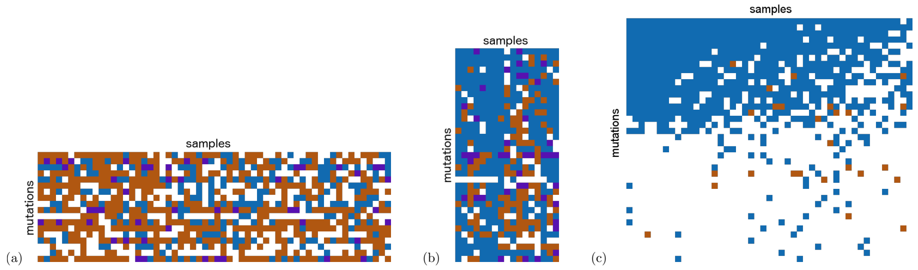

Fig. S1: Mutation matrices used in this study. (a) JAK2-negative myeloproliferative neoplasm: single-cell exome sequencing of 58 tumor cells, 18 mutation sites selected for importance out of 712 SNVs [1]. Estimated error rates: 0.4309 for allelic dropout and 6.04×10^{-6} for false discovery, 45% missing data points. The mutation matrix is taken from [2]. Color coding of matrix cells: blue - heterozygous mutation called in cell, purple - homozygous mutation called in cell, white - site not mutated in cell, brown - missing data point; (b) Clear cell renal cell carcinoma: single-cell exome sequencing of 17 tumor cells, 35 mutation sites are not always present among the 50 detailed in the supplementary material of [3]. Estimated error rates: 0.1643 for false negatives and 2.67×10^{-5} for false positives, 22% missing data points. Color coding of matrix cells: blue - heterozygous mutation called in cell, purple - homozygous mutation called in cell, white - site not mutated in cell, brown - missing data point; (c) Oestrogen-receptor positive (ER^+) breast cancer: single nuclei exome sequencing of 47 tumor cells, 40 SNVs shared by at least two cells [4]. Estimated error rates: $9.72 \pm 2.19\%$ for allelic dropout, and 1.24×10^{-6} for false discovery. Missing data points: 1.4%. Color coding of matrix cells: blue - mutation is present in cell, white - site not mutated in cell, brown - missing data point.

⋆ Equal contributors

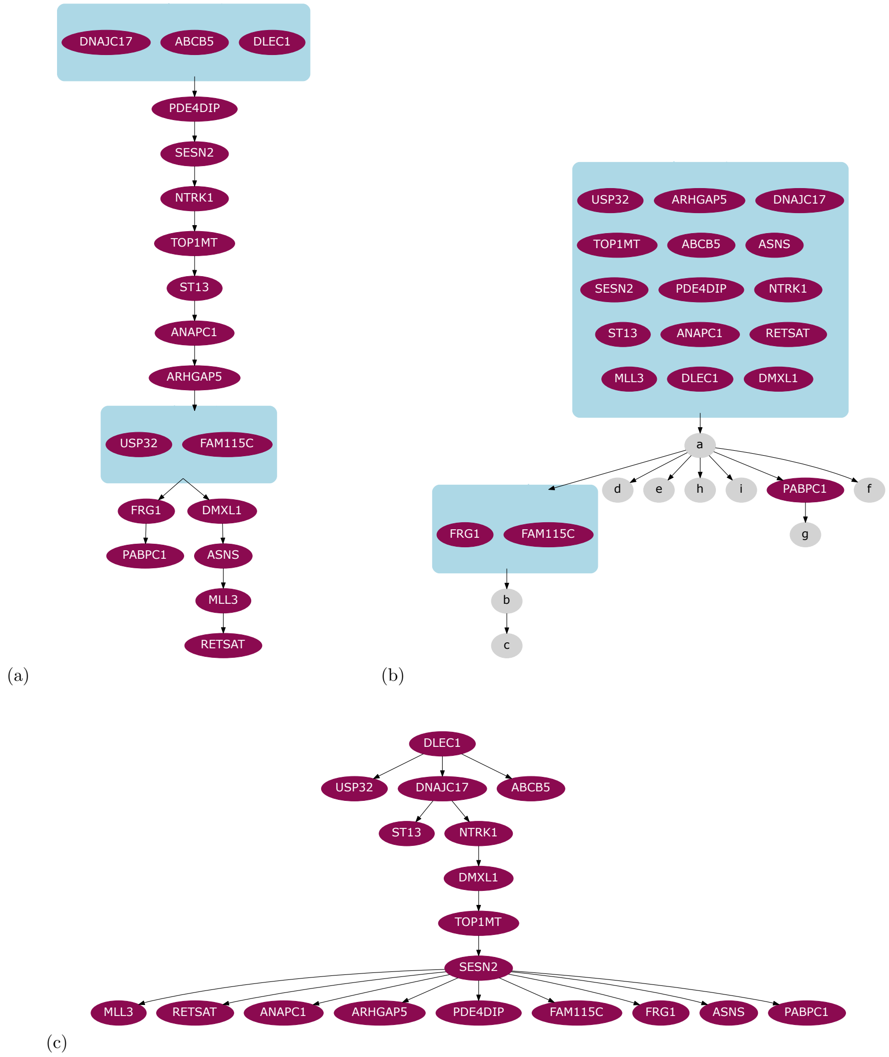

Fig. S2: Reconstructed trees for the JAK2-negative myeloproliferative neoplasm. (a) ML tree (for better comparability only the mutation tree without attached samples is shown here, see Fig. S3 for sample attachment). (b) Mutation tree built from the clonal tree inferred by BitPhylogeny [5]. Attached grey nodes are the subclones. (c) Mutation tree reconstructed by the approach of [2]. Blue boxes indicate mutation sequences with non-identifiable order

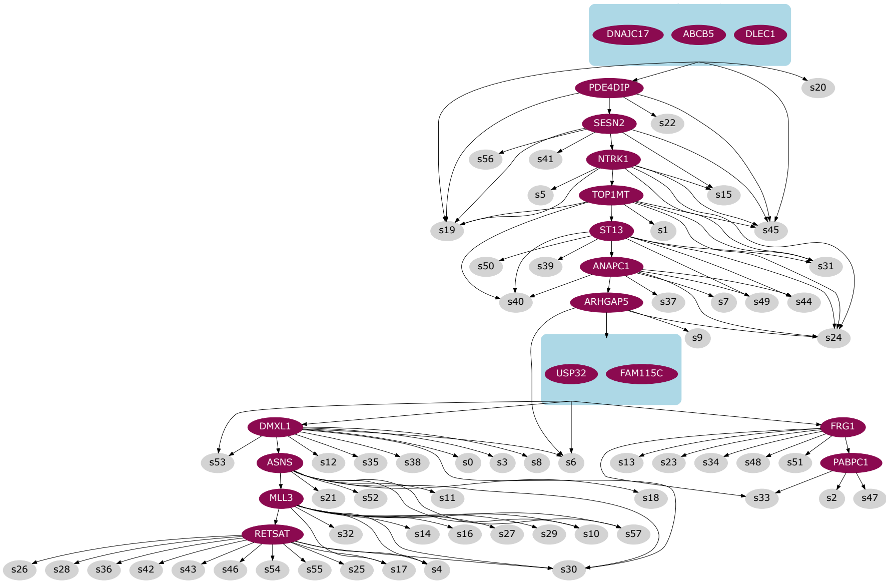

Fig. S3: ML tree with attached samples for the JAK2-negative myeloproliferative neoplasm. This is the complete version of the tree in Fig. S2(a) where sample attachment was omitted. Multiple parent edges pointing to a node indicate co-optimal attachment points. Discarding all but one of them for each node gives one ML tree.

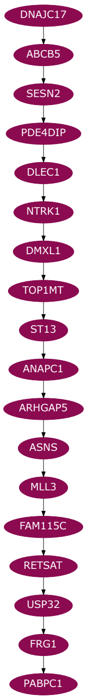

Fig. S4: The MAP tree for fixed and learned β for the JAK2-negative myeloproliferative neoplasm. The MAP tree obtained for learning false negative rate β of the Hou et al. data [1] using a beta prior with mean 0.4309 and standard deviation of 0.1 and a uniform tree and attachment prior. The same MAP tree is obtained for the fixed false negative rate of 0.4309, suggesting robustness of inference against small errors in the estimated error rates.

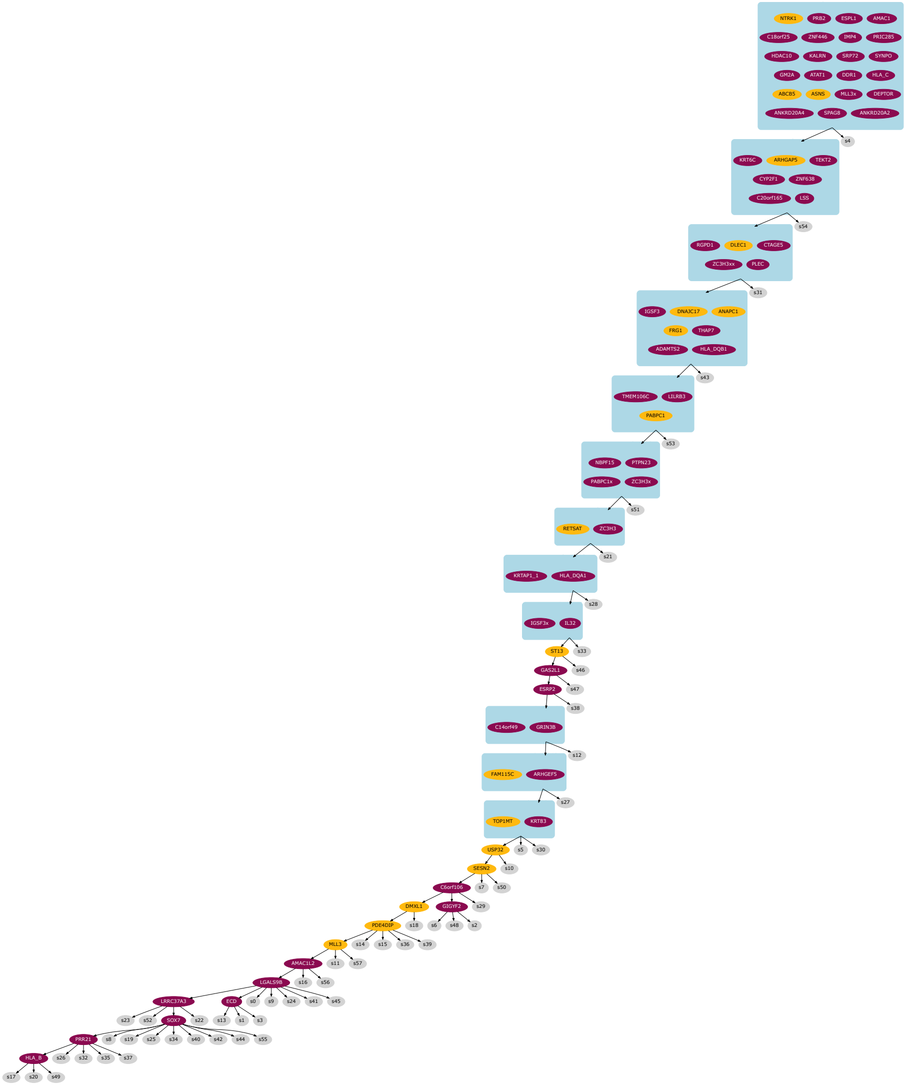

Fig. S5: ML tree for the JAK2-negative myeloproliferative neoplasm with 78 mutations. A ML tree with attachments for the Hou et al. data [1] when all 78 non-synonymous mutations are included. The tree has an overall linear structure as was the case when only 18 selected mutations highlighted in yellow were included (Figs. S3 and S4), but the order of mutations does vary. The order, however, is essentially determined by the smaller number of noisy samples placed higher up the tree.

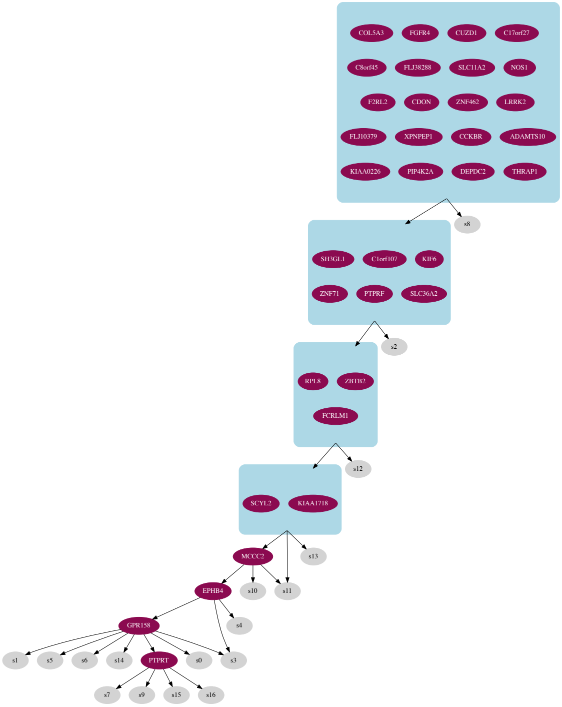

Fig. S6: ML tree for the clear cell renal cell carcinoma. An example ML tree with attachments for the clear cell renal cell carcinoma [3]. With the missing data and small number of samples there are many co-optimal trees, but they share a linear tree structure which suggests a monoclonal tumor progression. Blue boxes indicate mutation sequences with non-identifiable order.

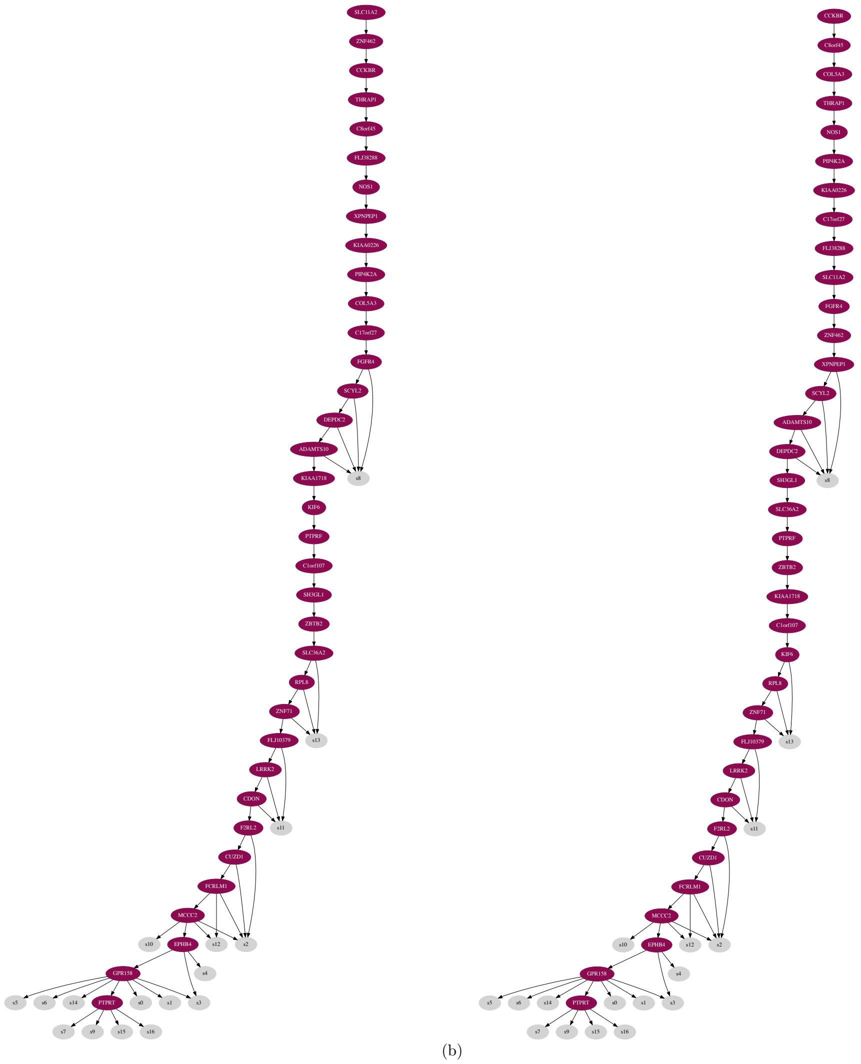

(a) (b)

Fig. S7: MAP trees for fixed and learned β for the clear cell renal cell carcinoma. Example MAP trees for the clear cell renal cell carcinoma [3]. For comparison, the samples are added to the MAP tree with their optimal placement. (a) With a fixed false negative rate β of 0.1643. (b) With learned β under a beta prior with mean 0.1643 and standard deviation 0.06. Although the MAP β value is higher at 0.198, the tree structure in both case is identical. Instead with the small number of samples fitting best lower down the trees the posterior is fairly insensitive to the order of mutations higher in the tree and this varies with differing values of β.

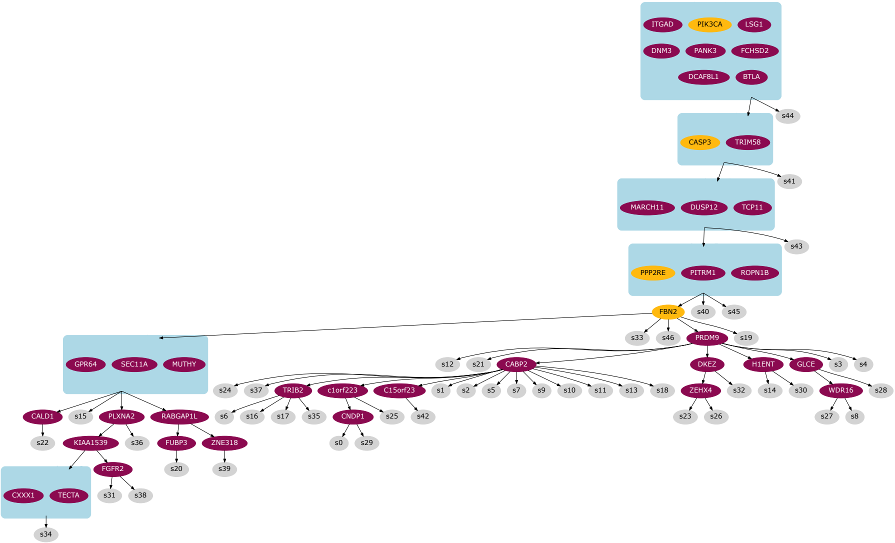

Fig. S8: ML tree for the (ER$^+$) breast cancer. The ML tree with attachments for the (ER$^+$) breast cancer dataset [4]. The tree structure suggests a monoclonal progression of the tumor in its early stages followed by the development of a complex subclone structure in more recent tumor stages. Yellow genes indicate non-synonymous mutations in known cancer genes [4]; blue boxes indicate mutation sequences with non-identifiable order.

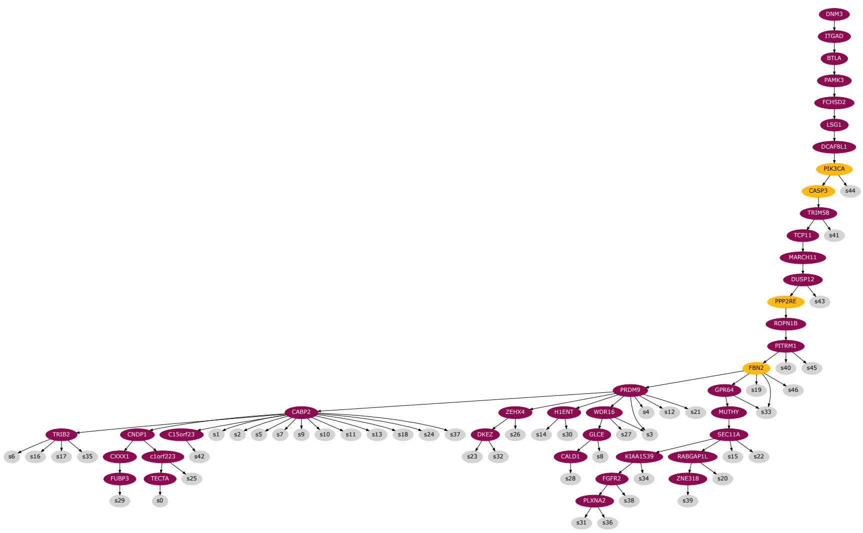

(a)

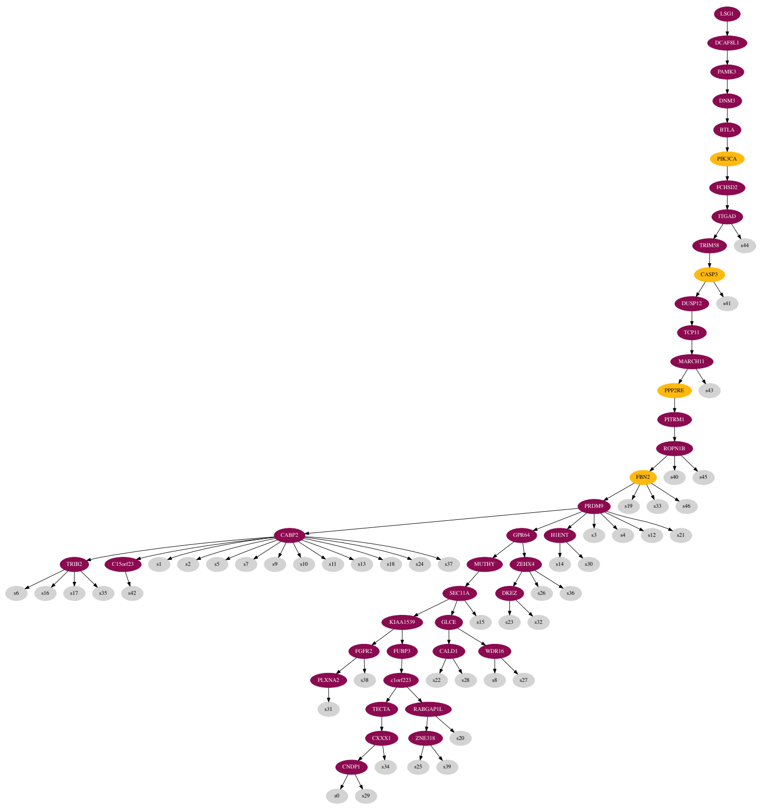

(b)

Fig. S9: MAP trees for fixed and learned β for the (ER^+) breast cancer. The MAP trees with uniform priors for the (ER^+) breast cancer dataset [4]. The samples were placed back in at the optimal attachment point after fixing the mutation tree. (a) With fixed false negative rate $\beta = 0.0972$. The topology is very similar to the ML tree optimized over mutation tree and sample placement in Fig. S8. (b) The tree when β is learned using a beta prior with mean 0.0972 and standard deviation 0.04. The MAP value of β is much higher at 0.227 resulting in a fairly different tree. There are strong similarities in the overall structure, with some rearrangements lower in the tree and some reordering of mutations higher up. Yellow genes indicate non-synonymous mutations in known cancer genes [4].

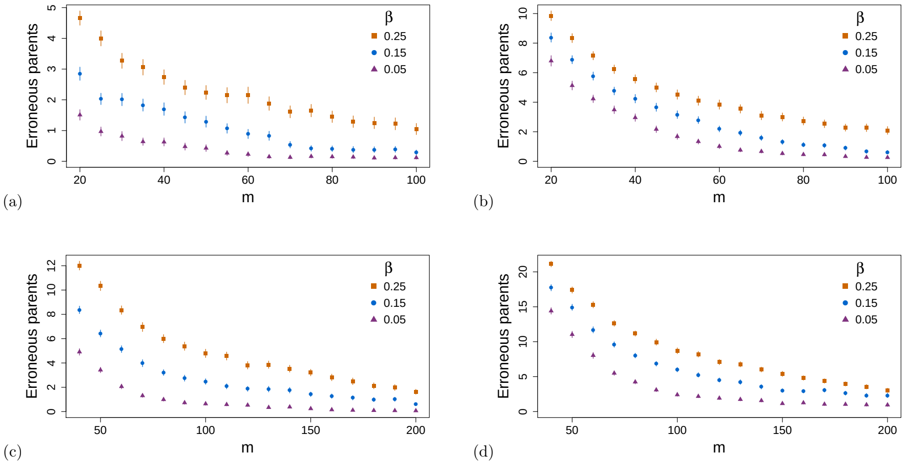

Fig. S10: Simulation of tree reconstruction. Average difference between the reconstructed tree and the true tree used to generate the data as the number of samples m is varied for three false negative rates $\beta = 0.05, 0.15$ and 0.25. The error bars are standard errors from the 100 runs. (a) ML tree for $n = 20$ (b) MAP tree for $n = 20$ (c) ML tree for $n = 40$ (d) MAP tree for $n = 40$. The ML tree distances are lower since they do not include non-identifiable regions.

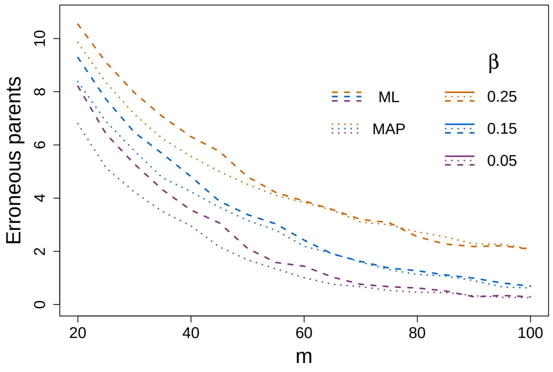

Fig. S11: Comparison of ML and MAP inference. Comparison of the tree learning for $n = 20$ using SCITE for the ML tree (dashed) and MAP tree (dotted). The settings are identical to the top row of Fig. S10 but to directly compare the two types of inference a random ordering is chosen for the non-identifiable regions in the ML trees. MAP inference performs slightly better.

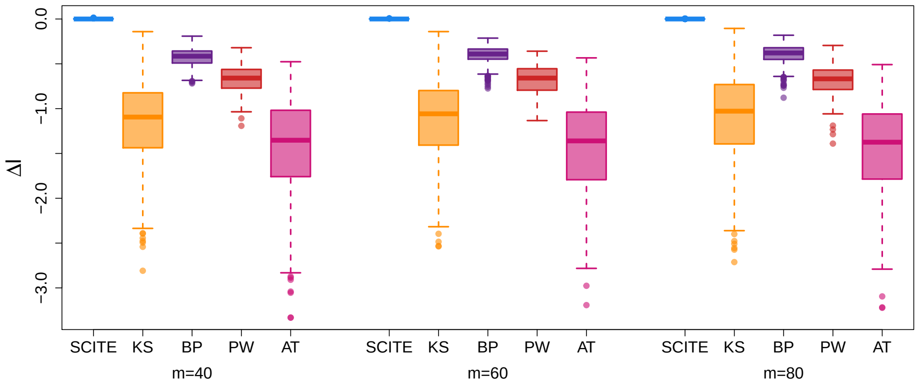

Fig. S12: Likelihood comparison of additional methods. Comparison of the tree inference of SCITE, the algorithm of Kim and Simon [2], BitPhylogeny [5], PhyloWGS [6] and AncesTree [7]. The quantity Δl is the normalized difference in log-likelihoods between the inferred and generating trees.

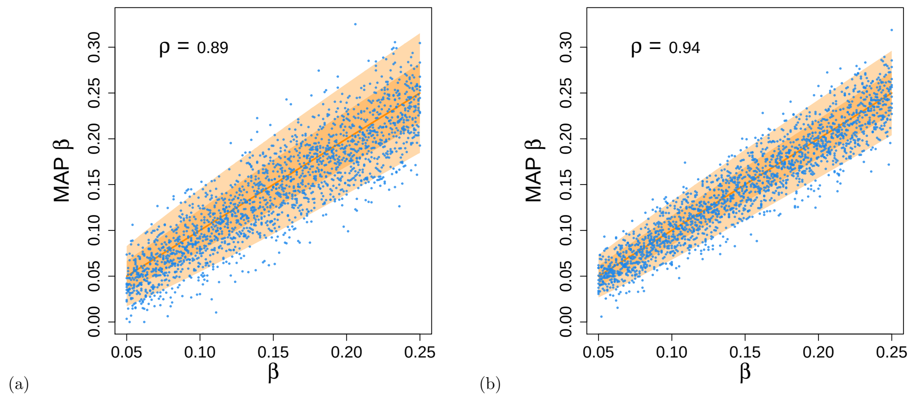

Fig. S13: Learning error rates. Comparison of the MAP β learned using SCITE for $n = 20$ against the false negative rate β used to generate the data. (a) For $m = 40$ (b) for $m = 80$. The solid blocks are one and two standard deviations of inferring β if the tree was known.

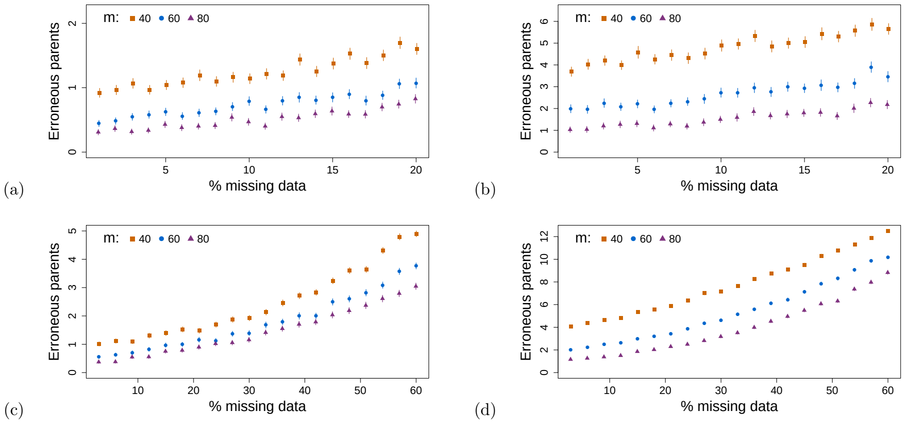

Fig. S14: Simulation of missing data. Average difference between the reconstructed tree and the true tree used to generate the data as the missing data rate is varied for $m = 40, 60$ and 80 samples with $n = 20$. In (a) and (b) we focus on missing data rates up to 20% while (c) and (d) cover higher rates up to 60%. ML tree inference is presented in (a) and (c) with no penalization for non-identifiability while (b) and (d) are with the MAP tree. The error bars are standard errors from 400 runs, except for (b) which is over 100 runs.

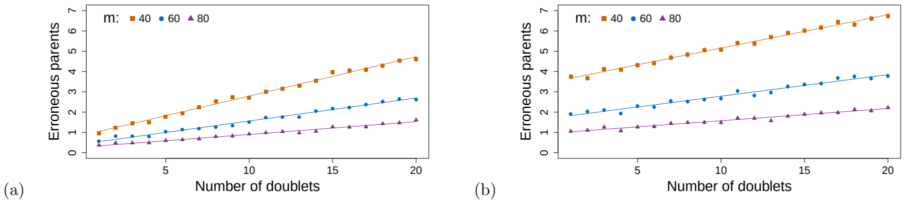

Fig. S15: Effect of doublet samples. Average difference between the reconstructed tree and the true tree used to generate the data as the number of doublet samples is increased out of the $m = 40, 60$ and 80 total samples. (a) ML tree for $n = 20$ (b) MAP tree for $n = 20$. The error bars are standard errors from 400 runs. Non-identifiable regions are not included in the ML distances.

Supplementary Tables

Table S1: Comparison with perfect phylogeny. The average number of erroneous parents in tree reconstruction from noisy data matrices for classic perfect phylogeny reconstruction (PP), our maximum likelihood (ML) and maximum a posteriori (MAP) tree reconstruction. Setting are $n = 20$, $m = 20, 25, 30, \ldots$, $\beta = 0.05$, and 100 matrices were generated for each value of m. The error rates are averaged over only those instances where a perfect phylogeny exists (mutation matrices with no contradicting data points). The fraction of contradiction free matrices for each m is given in the last row. While the experiment ran up to $m = 100$, the table is truncated after $m = 45$ as no contradiction free matrices were generated for $m > 45$.

	# samples m					
	20	25	30	35	40	45
avg. # erroneous parents in PP	6.5	5.75	6.0	4.5	3.5	3.0
avg. # erroneous parents in MAP	3.9	3.25	4.5	3.5	3.5	3.5
avg. # erroneous parents in ML	0.9	0	2.5	2.5	2.5	2.5
% of data sets with no contradiction	10%	4%	2%	2%	2%	2%

Supplementary Material

Additional moves in the MCMC of SCITE

To speed up the convergence of the chain we use two additional moves in our MCMC scheme.

Swap node labels. This move samples two of the n nodes uniformly and transposes them. While $q(T, \boldsymbol{\theta}|T', \boldsymbol{\theta}) = q(T', \boldsymbol{\theta}|T, \boldsymbol{\theta})$ simplifying Equation (14) of the main text, this move is neither irreducible nor necessarily aperiodic. But by including this on top of a chain built from the *prune and reattach* move we inherit those properties. In fact we swap two node labels with some fixed probability so we do not need to combine the two moves into a single neighborhood.

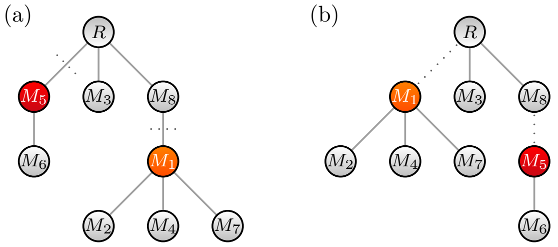

Fig. S16: Simple swap subtree MCMC move. (a) From our starting tree T we select two different nodes uniformly, here numbers 5 and 1, and detach them from the rest of the tree. (b) Then we swap the detached subtrees and reattach them. To reverse the process we would again choose nodes 1 and 5.

Swap subtrees. For this third move, we select two of the n nodes uniformly. If the two nodes, i and k, are not in an ancestor/descendant relationship we detach them from their parents and reattach them to each others' former parent (Fig. S16). Since for the reverse move we would simply need to select the same pair of nodes, $q(T, \boldsymbol{\theta}|T', \boldsymbol{\theta}) = q(T', \boldsymbol{\theta}|T, \boldsymbol{\theta})$ and calculating the acceptance probability in Equation (14) of the main text reduces to the ratio of tree scores.

In the other case, assume k is a descendant of i. First we can cut the edge leading to k and move it with its subtree and attach it to the parent of i. When we next detach i and its remaining subtree (with k and its descendants removed) to make the move reversible we need to sample the new parent of i from among k and all of its descendants, which we do uniformly. To reverse the move, we again need to sample i and k at the start and also to sample the previous parent of k from among i and its new (remaining) descendants. If we denote the number of descendants of i as $\mathrm{d}(i)$, the proposal probabilities now depend also on $\mathrm{d}(i)$ and

d(k), and the ratio of the forwards and backwards probabilities is simply

$$\frac{q(T, \boldsymbol{\theta}|T', \boldsymbol{\theta})}{q(T', \boldsymbol{\theta}|T, \boldsymbol{\theta})} = \frac{\mathrm{d}(k) + 1}{\mathrm{d}(i) + 1}$$

which we use in Equation (14) of the main text along with the ratio of tree scores. This move is illustrated in Fig. S17.

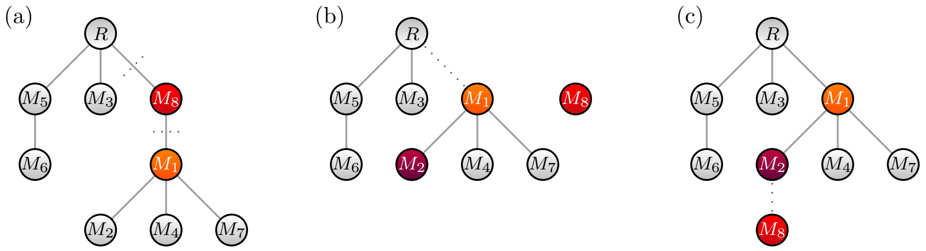

Fig. S17: Nested subtree swap MCMC move. (a) From our starting tree T we select two different nodes uniformly, here numbers 8 and 1. Since node 1 is a descendant of node 8 we detach node 8 from the main tree then attach node 1 and its subtree to the previous parent of 8 to arrive at (b). Node 1 or one of its descendants are chosen uniformly, here node 2 is picked. (c) This becomes the new parent of node 8. To reverse the process we would again choose nodes 1 and 8.

Combining the three MCMC moves. We build a mixture MCMC scheme where we pick one of the three moves at each step of the chain with a fixed probability. (In our examples we chose probability 0.1 for *prune and reattach*, 0.65 for *swap node labels* and 0.25 for *swap subtrees* as this gave good performance for the linear tree from the Hou et al. data [1].) Since the prune and reattach move satisfies the required properties of an MCMC scheme, the mixture similarly allows us to sample mutation trees according to their posterior. Now however we may do so more quickly due to the additional moves.

Performance gain by combining moves. To find out how much the additional moves can improve performance we used the same 400 trees with 20 nodes generated for the comparison of different methods with $m = \{40, 60, 80\}$. For each tree we found the average number of steps needed by SCITE to find a ML tree as in the main text as we varied the probability of each move in steps of 0.05 from 0.05 to 0.9. There is a large region with similarly good performance indicated by the purplish basins in Fig. S18. These regions consist of high and roughly equal amounts of the *prune and reattach* and *swap node labels* moves and low levels of the third *swap subtrees* move. The optimal probabilities for the simulated data are around $(0.55, 0.4, 0.05)$ respectively which provides a speed

up of a factor of 3 or 4 compared to using choosing the *prune and reattach* move 90% of the time, increasing to a factor of 17–19 compared to choosing *prune and reattach* with 98% probability. This indicates the scale of improvement that allowing the additional moves, especially swapping node labels, provides.

The optimal probabilities also find the ML tree around 2 or 3 times faster compared to the choice of $(0.1, 0.65, 0.25)$ above for such simulated data.

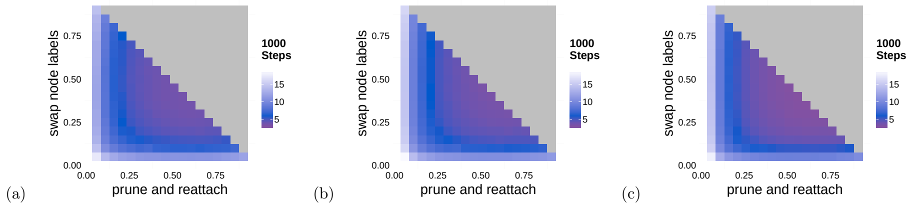

Fig. S18: MCMC move probabilities. The average number of steps taken for SCITE to first find a ML tree as the probability of the three MCMC moves is varied. The trees have $n = 20$ mutations with $m = 40$ attached samples in (a), $m = 60$ in (b) and $m = 80$ in (c).

Effect of γ on convergence. Along with the move probabilities, we may also vary the parameter γ which flattens or amplifies the score landscape, making the moves easier or harder to accept. With the optimal move probabilities there is a basin of γ values with good performance around the optimal value just below $\gamma = 1$ [Fig. S19(a)]. Performance starts to degrade quickly for values which are too small below $\gamma \approx 0.5$ and degrades more slowly for too large values above around $\gamma \approx 1.5$.

We observe similar behavior with move probabilities set to $(0.1, 0.65, 0.25)$ [Fig. S19(b)], suggesting that selecting $\gamma = 1$, as also required for the MCMC sampling, is a good choice.

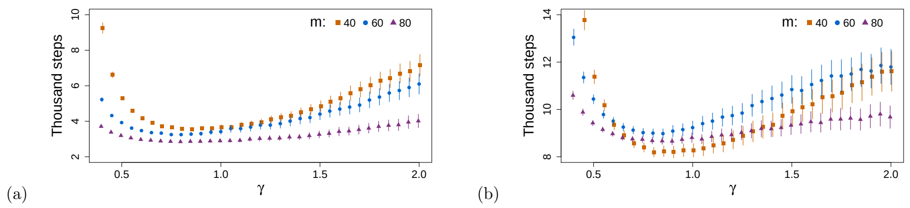

Fig. S19: MCMC parameter. The average number of steps taken for SCITE to first find a ML tree with $n = 20$ mutations as the parameter γ is varied. With approximately optimal move probabilities $(0.55, 0.4, 0.05)$ in (a) and move probabilities $(0.1, 0.65, 0.25)$ in (b).

References

[1] Hou, Y., Song, L., Zhu, P., Zhang, B., Tao, Y., Xu, X., Li, F., Wu, K., Liang, J., Shao, D., *et al.*: Single-cell exome sequencing and monoclonal evolution of a JAK2-negative myeloproliferative neoplasm. Cell **148**, 873–885 (2012)

[2] Kim, K.I., Simon, R.: Using single cell sequencing data to model the evolutionary history of a tumor. BMC Bioinformatics **15**, 27 (2014)

[3] Xu, X., Hou, Y., Yin, X., Bao, L., Tang, A., Song, L., Li, F., Tsang, S., Wu, K., Wu, H., *et al.*: Single-cell exome sequencing reveals single-nucleotide mutation characteristics of a kidney tumor. Cell **148**, 886–895 (2012)

[4] Wang, Y., Waters, J., Leung, M.L., Unruh, A., Roh, W., Shi, X., Chen, K., Scheet, P., Vattathil, S., Liang, H., *et al.*: Clonal evolution in breast cancer revealed by single nucleus genome sequencing. Nature **512**, 155–160 (2014)

[5] Yuan, K., Sakoparnig, T., Markowetz, F., Beerenwinkel, N.: Bitphylogeny: a probabilistic framework for reconstructing intra-tumor phylogenies. Genome Biology **16**, 36 (2015)

[6] Deshwar, A.G., Vembu, S., Yung, C.K., Jang, G.H., Stein, L., Morris, Q.: PhyloWGS: Reconstructing subclonal composition and evolution from whole-genome sequencing of tumors. Genome Biology **16**, 35 (2015)

[7] El-Kebir, M., Oesper, L., Acheson-Field, H., Raphael, B.J.: Reconstruction of clonal trees and tumor composition from multi-sample sequencing data. Bioinformatics **31**, 62–70 (2015)
